# Clinic of Professor Dunglison

**Published:** 1845-02

**Authors:** Samuel G. White

**Affiliations:** Georgia


### CLINICAL LECTURES AND REPORTS.

### PHILADELPHIA HOSPITAL.

*Saturday, December* 14, 1844.

### CLINIC OF PROFESSOR DUNGLISON.

Reported by Mr. Samuel G. White, of Georgia.

To report progress, the patients who had on several previous occasions been under consideration were brought before the class.

Of these: the first was the female with TYPHOID FEVER—she has entirely recovered, and is at present under no treatment except such means as are necessary to keep open the bowels.

The man, who suffered with SUBACUTE RHEUMATISM, had also much improved, the affection of the joints having disappeared, and the tumours, with which it will be remembered the case was complicated, diminished. The treatment required at present, with the exception of the use of the sulphate of quinia, of which he takes two grains three times daily, is strictly of a temporizing character. The opinion stated in the last lecture, as to the neuropathic nature of rheumatism, seems supported by the effect of remedies employed in this case,—being almost entirely of a tonic kind; yet in such rheumatic affections the augmentation of the fibrinous constituent of the blood is often as high as ten parts in the hundred, and this was probably the proportion in the present instance.

The lecturer here remarked, that in such cases the abstraction of blood does not diminish the proportionate quantity of fibrin, but rather the number of red corpuscles contained in the blood.

As the cupped state and buffy coat of the blood is dependent on the amount of fibrin, it can be readily comprehended—as this constituent suffers no diminution from bloodletting—that it is not necessary or philosophical, as was formerly supposed, to continue the use of venesection so long as the blood presents these appearances. A thorough conviction of this fact will often prevent the adoption of a plan of treatment, not only useless, but productive of injury.

The attention of the class was next directed to two cases of

INTERMITTENT FEVER, a disease extremely common in many portions of this country, as it is elsewhere. In the majority of cases it is so mild as to be easily managed by old women, without the aid of the physician; nay, in some "aguish districts," the fact of an individual having a chill is considered an insufficient excuse to exempt him from the duties of a juryman.

Most of the cases of this disease seen in this hospital are brought from a distance, although intermittents are frequently prevalent along the banks of the neighboring streams; and these are both vernal and autumnal. It is often questionable, however, whether agues occurring in the spring be not the result of exposure to its exciting cause during the previous autumn,—the malarious impression having remained dormant through the winter. The autumnal variety is most commonly met with.

Intermittents occurring in the spring, from their unusual mildness, are generally very easily managed, and from the supposition which it need scarcely be said is erroneous—that by them certain morbific matters are expelled from the system, which would otherwise have been injurious,—the old saying arose, that

An ague in the spring,
Is physic for a king.

The professor observed that it was not his intention to enter fully into the description of intermittents. He would merely allude to some of the prominent points connected with them. An intermittent consists of three stages, which constitute its paroxysm;—the cold stage, or stage of concentration, which is succeeded by the hot, or stage of expansion, and this again by the sweating stage, or stage of resolution. The period that elapses, from the termination of one to the commencement of another paroxysm, is the apyrexia or intermission; and the duration of this period constitutes the type of the disease. Thus, if there be a recurrence of the paroxysm every day, it is a quotidian; if every other day, a tertian; every third day, a quartan, &c. These may be double, and then we have what is termed a double quotidian, where there are two paroxysms in the space of twenty four hours; double tertian, where there is a daily paroxysm, those on alternate days corresponding; double quartan, where there is a paroxysm on two successive days, and then an interval of one day,—those occurring every seventy-two hours being alike, &c. In the same manner there may be triple tertians, &c.

The paroxysms may recur at the same hour every day, or there may be considerable irregularity in their recurrence, and when this is the case, they are more difficult to manage, inasmuch as the remedies cannot be directed with precision as to time, on which much depends. When the succeeding paroxysm comes on unusually soon, the intermittent is said to be "anticipating;" if not until after the usual time, it is a "postponing" intermittent. Inasmuch as the former is liable to run into remittent fever, it is more difficult to treat than the latter. The more regularity the paroxysms observe, the greater the facility with which they may be arrested.

As before stated, the professor did not intend to enter minutely into the pathology of intermittents, but he would allude to two points of much interest from their frequent occurrence—the enlargement of the spleen, and œdema of the lower extremities so often seen in this disease.

In the great majority of patients laboring under this form of fever, careful examination will detect some enlargement of the spleen. From this fact, it was inferred that the function of that viscus is to act as a diverticulum to the blood, which, by accumulating in its extensible tissue, was prevented from injuring more important parts. This enlargement of the spleen may be owing—as is the case where the disease is of short duration—to simple accumulation of blood or hyperæmia; or, as occurs in chronic cases, to hypertrophy consequent upon the modified nutrition induced by the frequent repetition of the engorgement. Sometimes the size of the viscus becomes enormous, extending far below the margins of the ribs.

From the depraved condition of the system, occasionally accompanying splenic disease, it has received in India the appellation of "splenic cachexia" in which there occurs hemorrhage from the stomach or bowels. Frequently after an attack of intermittent fever, a collection of fluid within some one of the great cavities supervenes; or, as more frequently happens, in the cellular tissue of the lower extremities, producing œdema. The effusion, or collection of fluid is consequent upon the irregularity of the circulation during the disease, which disturbs the balance between the exhalents and absorbents of the parts implicated.

The treatment of intermittent fever, as before remarked, is usually simple: often, indeed, the disease will pass away without any medicine being given; or it will seem to wear itself out, as was frequently witnessed some years ago, when the views of Broussais were prevalent. Intermittents were then treated as periodical inflammations of the gastro-intestinal mucous surface; for it will be remembered, that Broussais believed "every regular paroxysm of an intermittent is the sign of a gastro-enteritis." Yet some of the remedies employed are not such as we should think adapted for inflammation of the intestinal mucous membrane,—black pepper infused in whisky, for example.

Professor Dunglison considers intermittent fever to be largely neuropathic, and this view he founds on its cause, the periodicity of the paroxysms, and the effect of remedies. He believes the primary impression of the cause, malaria, to be made on the nervous system, and the cold stage to be a consequence of the depressing influence thus induced;—this being succeeded in regular order by the hot stage, or stage of reaction, and the sweating stage, in all of which the nervous system is probably materially concerned.

The idea of periodicity, he conceives, necessarily involves that of nervous influence. By what laws the periodical recurrence of agues are controlled little is known. Of this, as of other diseases characterized by periodicity, our ignorance is profound. The fact would seem to be, however, that there are, in the animal economy, periodical movements at different hours of the day corresponding with the movements of similar periods in other days, which may be concerned in the causation of the regular exacerbations noticed in intermittent, hectic fever, menstruation, &c. The effect of remedies likewise affords strong evidence of the neuropathic nature of the disease under consideration. They consist almost entirely of agents which produce their impression on the nervous system.

Again, it is well known, that any powerful emotion or excitement, mental or moral, is frequently sufficient to arrest a paroxysm. This is noticed, at times, in patients brought before the class in the amphitheatre. By practising the manipulations of the animal magnetizer, a paroxysm may be arrested; and the professor related a case mentioned to him by Ex-President Madison, in which Lafayette, who had been a pupil of Loutherberg,—himself a pupil of Mesmer,—at the suggestion of Mr. Madison, and in his presence, tried the effect of these manipulations on his servant, in whom the cold stage was commencing, and succeeded. In such cases, the new impression made on the nervous system is sufficient to break in upon the chain of morbid phenomena constituting the paroxysm. Upon this supposition may also be explained the effect of charms, and similar means that excite the imagination, in arresting ague.

The lecturer believes, that all the remedies employed with the view of checking the paroxysm act principally on the nervous system. It is very probable, that this is the sole action of the preparations of quinia, and to a great extent of cinchona, although others of its constitutents may have an influence. Whenever there is a want of tone resulting from any cause—except where there is a broken down state of the solids, as in scorbutic cachexia, —the depressing influence is exerted almost wholly on the nervous system. An excellent exemplification of this is found in the influence on the same system of varying conditions of the atmosphere. When the air is dry and cold, there is great buoyancy of spirits, a feeling of "corkiness" as it is termed by the trainers; but on the other hand, if it be damp, and loaded with moisture, there is great depression of spirits, languor and lassitude.

In some of the congestive forms of intermittents, unless a new impression is speedily excited, so as immediately to break in upon the disease, reaction cannot be established. In such cases, venesection, as proposed by Armstrong in congestive typhus, may be resorted to, combined or not with the hot bath. Bloodletting practised during the cold stage of an intermittent, is not, as might appear at first view, unphilosophical; for if it be recollected that during this stage there is a great centripetal tendency of the blood, and consequently congestion of the internal viscera, it can be readily comprehended, that venesection, by diminishing the amount of the circulating fluid, and exciting a reflux towards the bleeding orifice on the surface, may enable the organs better to unload themselves, and may thus be highly beneficial. This is an example of revulsive bleeding; but it should be carried to a slight extent only, or it may be positively injurious by depressing the powers. The moderate abstraction of blood is often productive of the most happy effects, and by it the length of the paroxysm may not only be diminished, but the disease be rendered more manageable. It can also be readily conceived, that the excitement of the cutaneous capillaries induced by the hot bath, may aid the supervention of the stage of expansion in highly congestive cases.

In every case of intermittent, however, the great reliance of the practitioner is placed in the sulphate of quinia, or some preparation of cinchona. The professor's plan of treating an intermittent; when the paroxysms recur regularly, is the following. He inquires, first of all, into the condition of the intestinal canal; and if there should be any irritation consequent upon the presence of offending matters, he administers a gentle emetic or cathartic, or both, as the case may be, so as to prepare the mucous surface for the better reception of the quinia. When the doctrines of Broussais prevailed, these articles—emetics and cathartics—were sedulously avoided, under the supposition that they would augment the gastro-enteric inflammation presumed to exist. The milder emetics only are necessary, as ipecacuanha; but sometimes the antimonii et potassæ tartras may be demanded. Rhubarb and calomel, or jalap and calomel, may be needed as a purgative, or some of the other articles of the class of cathartics. Having thus prepared the mucous surface for the administration of the antiperiodics, he is in the habit of using this prescription.

| | | |
|---|---|---|
| ℞ Quiniæ Sulphatis, | gr. x. | |
| Acid. Sulph. dilut., | gtt. vj. | |
| Aquæ, | f.℥iv. | M. |

Half of this may be given one hour before the expected paroxysm, where the paroxysms occur regularly, and the remainder half an hour afterwards. This he has found, in the majority of cases, sufficient to arrest the attack. Should it fail, however, and the premonitory symptoms of ague come on,—cutis anserina, paleness of surface, lividity of nails, &c.—recourse may be had to the tinctura opii, of which fifty drops may be given immediately; and, under this combination of agents that impress the nervous system, the paroxysm is in almost all cases prevented. This, again, is a remedy whose action is prominently exerted on the nervous system, and its modus operandi in intermittents is readily explicable. On every consideration, the lecturer thinks, without any impropriety, intermittents might be dismissed from the class of fevers proper, and placed among diseases of the nervous system.

In some cases the sulphate of quinia fails to effect a cure, and then benefit may be derived from the liquor potassæ arsenitis, given alone; or 8 drops may be combined with each dose of the sulphate of quinia. Should there be irregularity in the recurrence of the paroxysms, the quinia cannot be given as above directed, but must be distributed through the apyrexial stage, as it is important to make the requisite impression sufficiently early to arrest the succeeding paroxysm; and if delayed until the last hour, it may return before the remedy can be effective.

Considerable discrepancy exists among practitioners as to the best mode of exhibiting the sulphate of quinia; some preferring to distribute it throughout the intermission; others to give it shortly before the expected paroxysm. The latter plan is preferred by the lecturer, although he thinks it unimportant in what manner it is used, if sufficient be given to produce the requisite impression before the paroxysm appears. In some instances, where the sulphate of quinia has failed to arrest the disease, the cinchona has been substituted with advantage. It is probable that its efficacy, in these cases, is in part due to the impression made by the woody fibre, although its other constituents may have some influence also.

The professor here exposed the side of one of the patients to point out the enlargement of the spleen. In this case, however, it was not very marked, having diminished considerably within a few days past. This was of the quotidian type, and the paroxysms had been arrested for several days. In the other patient the spleen was more enlarged. It could be felt distinctly below the ribs, and the dull sound on percussion was very marked. This was a case of tertian intermittent, but convalescence is now established.

It may be remarked, that in making an examination to detect the existence of enlarged spleen, it is desirable, that the patient should be in a recumbent position, with his left leg drawn up, so that the abdominal muscles may be relaxed.

The enlargement of the spleen usually passes away under the treatment necessary for the disease which gives rise to it. The ferri sub-carbonas has lately been highly lauded in such cases, and although it is generally unnecessary to give it, cases do arise which resist the sulphate of quinia, and it may then be resorted to with beneficial effects. In the cases now before the class, the sulphate of quinia will be continued, and if the size of the spleen should not diminish under its use, the subcarbonate of iron will be substituted for it.

Recently, in Paris, enormous doses of sulphate of quinia have been administered in the treatment of intermittent fever, but although it may have removed the disease, unpleasant encephalic accidents have been induced, and occasionally, it is asserted, death. But even if the practice of giving such large doses were devoid of danger, it is a species of extravagance in no way to be countenanced, for the same benefit may be obtained from a more moderate use of the article. There may be cases, however, in which, to effect a cure, the full narcotic and sedative influence of the sulphate of quinia may be required, and of course it is then warrantable to use large doses; but, as before stated, these are rarely met with; and in the vast majority of instances, the plan above indicated will be sufficient to arrest the disease.

The professor next introduced a patient to point out a single phenomenon connected with certain pulmonary affections, viz.:

JERKING RESPIRATION.

On examining the patient, who laboured under pharyngitis, the history of whose case has been previously detailed, it was noticed that the air passed into the lung in a jerking manner—a phenomenon which immediately excited attention. In this case it occurs under the left clavicle, but is not so marked as it sometimes is, and as it now is in a patient in the wards, but whom it would be imprudent to expose at this time. This variety of respiration has been pointed out recently as a valuable sign of phthisis in its incipiency, and is much dwelt upon by Dr. Walshe in his work on diseases of the lungs, which the lecturer considers one of the best manuals on the subject that have been published, and recommends it as a "vade-mecum" to the class. This jerking respiration is generally found in cases of tubercular depositions, and when present may assist much in the diagnosis. The patient under consideration has consolidation of the left lung near its summit, owing, in all probability, to a deposition of tuberculous matter,

The attention of the class was then directed to a case, of an affection decidedly neuropathic in its nature, viz.:

ASTHMA, not emphysema of the lungs; for although there is, in this latter affection, great difficulty of breathing, it is not of the spasmodic character which distinguishes nervous asthma, but is constant. Asthma principally attacks the adult and the aged; and when children at an early period of life suffer from chronic dyspnœa, it is in most cases dependent on emphysema of the lungs, and is not the disease under consideration.

Asthma, as remarked before, is a nervous affection, in which there is much difficulty of breathing, dependent upon a spasmodic contraction of the muscular portion of the air tubes, whereby their calibre is diminished. The lecturer considers, that the contraction of these muscular fibres is due to irritation, probably affecting, directly or indirectly, the pneumogastric nerves:—in other words, there is a state resembling cramp of the muscles, The vital manifestations of this disease are very distinct in most cases, there being much dyspnœa, wheezing respiration, and cough, accompanied or not by mucous expectoration.

As might be anticipated, no signal advantage is obtained from an examination by the physical methods—auscultation and percussion. There being no increase in the solidity of the pulmonary tissue, the percussion is not diminished in clearness. On applying the ear to the chest, a wheezing murmur is heard similar to that perceived at a distance, but much exaggerated, which is the consequence of the constricted condition of the tubes.

There may be an expectoration, with the cough, of frothy mucus, or there may be none, — the former constituting the *humid*, the latter the *dry* form of asthma.

Some practitioners, without any regard to the pathological state which constitutes the disease, abstract blood, which it need scarcely be said must generally be unnecessary. It should, in every disease, be the object of the physician first to discover the cause that gives rise to it, and then to adapt his remedial agents accordingly. This neglect of attending to the pathological condition is constantly witnessed in the treatment of persons attacked with urgent diseases, or suffering from accidents, as from a fall from a height, where, simply because there is loss of sensibility from injuries, venesection is immediately resorted to. The popular voice calls for the lancet, and the physician uses it without adequate reflection as to its propriety. In some instances venesection may be required in the treatment of asthma, and it must be employed. Usually, however, recourse is properly had to agents which act primarily on the nervous system; and of these, narcotics, as opium, are freely given, and their use continued until the paroxysm ceases. It is astonishing what immense quantities of opium may be taken with impunity in spasmodic diseases. The lecturer alluded to a recent case, seen by him with Professor Huston, in which there was spasm of the diaphragm, and which required very large doses of opium to overcome it; the patient having taken ℥ij. of the liq. morphiæ, and 180 drops of laudanum; but notwithstanding this quantity had been given, it was continued at intervals until the cramp ceased.

Many practitioners are in the habit of resorting, in asthma, to a class of medicines ranked as 'antispasmodic,' but there is in truth no remedy which is antispasmodic under all circumstances. The virtues of the class are dependent upon the existing state of the system, and if they remove that state they will relieve the spasm. The whole of the so called 'antispasmodics' produce their effect mainly by the new impression which they excite in the nerves of the mouth and stomach, which is sufficient to break in upon the morbid phenomena constituting the spasm. This is especially the case in hysteria, where the most nauseating substances are administered, which powerfully impress the mind, taste, and stomach of the individual.

In the true spasms of the muscles, that resist the efforts of the surgeon in the reduction of a luxation, 'antispasmodics,' so called, are never thought of; but he relaxes them by the proper employment of sedatives, as the lancet, or the internal use of tartrate of antimony and potassa, &c. In cramp of the gastrocnemii muscles we never resort to antispasmodics; and if not beneficial in ordinary spasm, why should they be expected to prove so in spasms of the muscular portion of the bronchial tubes?

The patient under consideration has suffered from bronchitis, but has recovered. The oleum tiglii, with a view to its revellent action, has been rubbed over the surface of the chest, and has produced a copious crop of pustules. The professor remarked that it was important for the class to be well acquainted with the appearance of these artificial eruptions, inasmuch as in the absence of a history of the case, they have been mistaken for cutaneous eruptions originating in a morbid condition of the system. The duration of a paroxysm of asthma is very variable, but usually it lasts for many hours; and, as before stated, the remedies must be continued until it is arrested.

The professor then proceeded to a few observations on some interesting

PATHOLOGICAL SPECIMENS, obtained from a patient who presented, when first seen, the phenomena of typhoid fever.

In all febrile affections, especially those in which there is much prostration of the vital powers, there is a liability to inflammation of the pulmonary tissue, known as INTERCURRENT PNEUMONIA. Sometimes the symptoms indicating its existence are extremely obscure, or even completely latent; but whenever—as remarked on a former occassion—there is much heat of surface, with dyspnœa and cough, it may be suspected, and examination of the physical signs may detect it. Inflammation of the terminal air-tubes may exist without pain. It may be accompanied with an expectoration of a mucous matter, with some dyspnœa; and as the disease proceeds, the sputa alter in character, becoming tenacious and of a rusty colour, with the increase of the other symptoms; and at a still later period, when the affection has proceeded to interstitial suppuration, the expectoration resembles, at times, prune juice. The sputa, however, may vary greatly in their appearance.

In the early stage of pneumonia, there is simple engorgement of the lungs, or, in other words, hyperæmia. If this continues, an effusion of the liquor sanguinis with some of the red corpuscles occurs, constituting the second stage, or that of red hepatization or consolidation, which may pass away, or run on into the stage of yellow or grey hepatization or of infiltration of pus.

The effusion that occurs in this last stage may consist of two kinds of matter; one, which is first thrown out, consisting of exudation corpuscles, being in contact with the lung membrane, and capable of some degree of organization ; the other, or unorganizable matter, on the surface of the former, and exposed to the air, by which it is decomposed. The two varieties of pus corpuscles constituting these have been distinguished by the terms, *cacoplastic*, which is applicable to those nearest the lung membrane, and unexposed to the air, and capable of an inferior grade of organization; and *aplastic*, or those entirely devoid of plastic or formative powers, and so situate as to be on the surface of the former, and exposed to the air, and in part constituting pus.

[To elucidate these remarks, the Professor introduced a diagram on the black-board, to exhibit the relative position of the two kinds of corpuscles in the suppurative process.]

When the stage of gray hepatization has fully formed, the expectoration again undergoes a change, and becomes purulent in character, and often, as before remarked, of a prune juice colour.

These are the stages of pneumonia usually described, but Dr. Stokes contends that before the stage of engorgement takes place, there is one in which there is an arrestation of the natural secretions, with a dry cough, and indicated by no marked physical phenomena.

Whenever the vital manifestations of pneumonia are obscure, and there is merely a suspicion of its existence, recourse is advantageously had to the physical signs, and here we have excellent evidence of their great utility. These signs, as might be expected, vary materially in the different periods of the disease. In the *first* stage of Stokes, as before remarked, there are perhaps no characteristic phenomena. The *second* stage is also unaccompanied by any marked signs of disease, except it be the very peculiar one known as the 'crepitant rhonchus,' which strikingly resembles the sound elicited by rubbing a lock of hair between the fingers, near the ear. The lecturer thinks that without impropriety we might consider pneumonia to be an inflammation of the lining membrane of the terminal tubes plus the cellular tissue connecting them. Admitting this to be the case, we have laryngitis or inflammation of the larynx ; tracheitis, or that of the trachea;—bronchitis, or inflammation of the larger tubes; and lastly pneumonia, or inflammation of the terminal branches of the same tubes plus the inter-bronchial texture itself,—as inflammations in different portions of the same tubes. The crepitant rhonchus might be supposed to be produced by the bursting of minute bubbles of air, passing through the viscid mucus in these tubes. Sometimes in the smaller tubes there is a *râle*, bearing some resemblance to the crepitant, called the 'sub-crepitant,' but which is not a sign of pneumonia. It is only the crepitant, or that produced in the terminal cells, that is so characteristic of the disease. It is determined that the mucus rhonchus is produced by the passing of air through mucous contained in the bronchia of larger size, but there is much doubt as to the true pathology of the crepitant rhonchus. It has been supposed by some, that it is caused by the passage of the air through blood effused into the terminal cells of the bronchia; but unless we can conceive that the blood adheres to the sides of the tubes, it is difficult to comprehend how the air can so pass through the blood, as to cause these phenomena; for if it be collected at the bottom of the tubes, it is manifest that air cannot readily enter it so as to form bubbles. The professor is disposed to think, that the crepitation is produced by the disruption of the glutinous lymph effused into the inter-cellular tissue, although he believes the true pathology is not yet thoroughly understood.

In the second stage of pneumonia, or that of red hepatization, the physical phenomena are more marked, and can easily be inferred from a knowledge of its anatomical condition. In it, there is consolidation of the pulmonary tissue, and consequently percussion elicits a flat sound : the pressure of the consolidated lung obliterates the smaller tubes, so that the respiratory murmur becomes extinct, and in fact no respiratory sounds are audible, except those produced in the larger tubes, constituting what is called 'bronchial or tubal respiration.' As the tissue, by being increased in density, becomes a better conductor of sonorous vibration, there is an increase in the vocal and tussive resonance, constituting 'bronchophony.'

This stage passes into that of gray hepatization, or purulent infiltration, and the physical signs remain nearly as in the last stage. When there is, however, a collection of pus, and the formation of a cavity, there are, in addition to the signs mentioned as belonging to the last stage, cavernous respiration and gurgling. Sometimes, when the excavation is large, the voice appears to pass directly into the ear; or there is 'pectoriloquy,' When the case is to terminate favourably there is a return of the crepitant *râle*, which had disappeared,—thence called *râle crépitant de retour, or rhonchus crepitans redux.* Of course, this indicates a resolution of the disease, and is accompanied by improvement in the general symptoms.

Sometimes the lung, after death, is simply engorged with blood, which has transuded from the vessels; and this is frequently observable in diseases that offer obstructions to the pulmonary circulation,—those of the heart especially.

The treatment of pneumonia, the lecturer remarked, would be the subject for future consideration, but he would observe that the cases seen in hospitals will not usually bear as active depletion as elsewhere. In ordinary cases, much good may be obtained from the contra-stimulant action of the tartarized antimony. It may be given in doses of 6 grs. in the course of the day, gradually increasing until it amounts to 24 grains in the day, and even more, should it be necessary. The system, after some doses of this medicine have been given, seems to acquire a great degree of tolerance to it, and the quantity can be rapidly increased. At times, however, the tolerance does not exist, and the remedy has to be discontinued. Blisters are also much employed, but not in the active stages of the disease: they are principally indicated in the latter periods. In cases of intercurrent pneumonia, venesection to a great extent cannot usually be practised, but revellent topical bleeding by cups may be very advantageous. In true typhoid cases, it is very beneficial to induce the revellent action of mercury on the system, which may be effected by the internal use of calomel. When there is a difficulty in producing the mercurial impression, the surface of a blister may be dressed with the unguentum hydrargyri. This plan of treatment the professor has found exceedingly beneficial in many cases.

After these general observations, the lecturer reverted to the case which had furnished the specimens before him. A man aged 33, entered the wards with many of the phenomena of typhoid fever, which, in the course of a day or two, were complicated with those of intercurrent pneumonia, as indicated by the ordinary functional phenomena, and physical signs. Consolidation of the pulmonary tissue rapidly supervened, and notwithstanding the employment of appropriate treatment, he died in a few days after the first evidences of pneumonitis.

On an examination of the body made 18 hours after death, the left lung was found consolidated, and sank readily in water; the upper and lower lobes being in a state of red hepatization, or, in other words, in the first stage of consolidation. The right lung was also engorged with blood; and both lungs had their several lobes united into one mass by pleuritic adhesions. The pericardium was joined to the lungs in a similar manner. The pulmonary tissue in the consolidated parts was readily broken down by the finger.

These appearances were exhibited to the class, accompanied by elucidatory remarks.

The lecturer reminded the class, that he had promised to have the lungs of the patient whose case had been described to them, as one probably OF GANGRENE OF THE LUNG, (*Examiner*, Jan. 1845, p. 20,) more carefully examined than they had been at the last lecture, in order to detect whether any gangrenous slough existed which might have previously escaped detection. This had been done by Dr. Farquharson, one of the resident physicians attached to his wards, who reported the following appearances. On a farther examination in the vicinity of a small cavity, and occupying a considerable portion of the middle lobe of the right lung, was a portion of pulmonary tissue, softer than the surrounding or other portions of the lungs. It was of a lighter colour than the inferior or more congested parts of the lungs, but darker than the apices, or portions of a nearly normal colour. The fluid which exuded upon pressure was not frothy, corresponding in colour with the part from which it came, and had an offensive odour, which was found to be decidedly different from that of any other portions of the same lungs, and also from that of other lungs of nearly the same age. The smell was peculiar, being fishy or that of putrid fish.

This portion of the lung was probably the seat of the fetid expectoration, which is so characteristic of a gangrenous condition of the lung.

The same gentleman had also further examined the lungs of the woman who had died of PHTHISIS ACCOMPANIED BY HÆMOPTYSIS, (*Examiner*, p. 19,) to detect, if practicable, the vessel or vessels whence the hemorrhage had proceeded. Although several vessels were found traversing the indurated mass at the apex of the right lung, no patulous orifice was found in the parietes of the cavity, which were examined after repeated washing in water. A large, apparently venous vessel, passed within a line or two of the cavity for the space of half an inch. Through the parietes of this vessel—the lecturer remarked—the transudation of blood might have taken place, but he is more disposed to think that it proceeded from vessels in the lower portion of the lung, which became engorged in consequence of mechanical impediments to the circulation in the consolidated portions,—the blood being poured into the neighbouring bronchia, and not into the tuberculous cavity.

